# Contribution of Recipient-Derived Cells in Allograft Neointima Formation and the Response to Stent Implantation

**DOI:** 10.1371/journal.pone.0001894

**Published:** 2008-03-26

**Authors:** Xiaoli Ma, Benjamin Hibbert, Dawn White, Richard Seymour, Stewart C. Whitman, Edward R. O'Brien

**Affiliations:** Vascular Biology Laboratory, University of Ottawa Heart Institute, Ottawa, Ontario, Canada; Leiden University Medical Center, Netherlands

## Abstract

Allograft coronary disease is the dominant cause of increased risk of death after cardiac transplantation. While the percutaneous insertion of stents is the most efficacious revascularization strategy for allograft coronary disease there is a high incidence of stent renarrowing. We developed a novel rabbit model of sex-mismatched allograft vascular disease as well as the response to stent implantation. *In situ* hybridization for the Y-chromosome was employed to detect male cells in the neointima of stented allograft, and the population of recipient derived neointimal cells was measured by quantitative polymerase chain reaction and characterized by immunohistochemistry. To demonstrate the participation of circulatory derived cells in stent neointima formation we infused *ex vivo* labeled peripheral blood mononuclear cells into native rabbit carotid arteries immediately after stenting. Fourteen days after stenting the neointima area was 58% greater in the stented *vs*. non-stented allograft segments (p = 0.02). Male cells were detected in the neointima of stented female-to-male allografts. Recipient-derived cells constituted 72.1±5.7% and 81.5±4.2% of neointimal cell population in the non-stented and stented segments, respectively and the corresponding proliferation rates were only 2.7±0.5% and 2.3±0.2%. Some of the recipient-derived neointimal cells were of endothelial lineage. The ex vivo tagged cells constituted 9.0±0.4% of the cells per high power field in the stent neointima 14 days after stenting. These experiments provide important quantitative data regarding the degree to which host-derived blood-borne cells contribute to neointima formation in allograft vasculopathy and the early response to stent implantation.

## Introduction

The artery wall consists of three layers: the (inner) intima, the media and the (outer) adventitia. Non-diseased human coronary arteries normally have a modest layer of intimal thickening (or neointima, NI) that consists of an accumulation of smooth muscle cells (SMCs) and extracellular matrix[1]. Currently, it is believed that medial SMC proliferation and inward migration, as well as the transmigration and retention of blood borne inflammatory cells play key roles in transforming a benign NI into the obstructive lesions that are seen in atherosclerotic coronary artery disease (CAD), as well as allograft coronary disease (ACD) that occurs after heart transplantation[2], [3]. Like garden variety CAD, ACD can ultimately lead to life threatening clinical sequelae, and the most common revascularization strategy for either of these entities involves the insertion of metallic scaffolding devices known as stents. While stent renarrowing due to recurrent NI formation (also know as in-stent restenosis or ISR) occurs in up to 5-30% of CAD lesions (depending on the stent and/or drug coating on the stent)[4], [5], ISR is a more frequent and serious problem for ACD lesions[3], [6]–[9]. For example, Simpson and colleagues report 6 and 12 month ISR rates of 41% and 53%; respectively, with 39% of stented patients dying or undergoing repeat cardiac transplantation within approximately 2 years of their first intervention[9]. The histopathology of ACD is characterized by inflammatory cell infiltrates with SMC accumulation and hence is more akin to ISR than the complex atherosclerotic lesions. Indeed, Jonas and colleagues recently demonstrated that angiographic progression of ACD and ISR lesions in allograft coronary arteries correlate and share histological features[3].

The pathogenesis of ACD is incompletely understood, and debate lingers over the relative importance of immune *vs*. non-immune factors, as well as the origin of NI cells. Unfortunately, experimental studies involving a variety of animal models of transplantation have produced discordant results (see review by Hillebrands *et al*.[10]). Indeed, there is critical concern when high-resolution confocal or deconvolution microscopy is not used to track cell lineage in allografts (see editorial by Hoofnagle *et al*.[11]). Regardless of these experimental studies, one cannot ignore observations from a handful of important human studies that suggest that host cells participate in allograft disease. For example, in 1971 Kennedy and Weissman[12] used anti-sera directed against donor and host HL-A antigens to suggest host cells were at least partially responsible for NI formation in a human cardiac allograft coronary artery. Together with at least five larger and more sophisticated studies of human transplantation, there is already irrefutable evidence that extracardiac cells engraft both non-diseased or diseased arteries of allografts[13]–[17].

Hence, while there is information available about the involvement of host-derived cells in allograft NI formation, many questions remain. For example, the precise degree to which host cells populate the allograft NI has only been assessed semi-quantitatively by sampling a relatively small number of cells in just a handful of arteries. Certainly, a more comprehensive quantitative assessment of the degree to which blood borne host cells participate in NI formation is required before we can assign a level of significance to these cells in the pathogenesis of allograft vascular disease, and determine the need to develop therapeutic strategies to inhibit their participation. Therefore, the goals of the current study are to address the following questions using a rabbit model of allograft vasculopathy and the response to stent implantation: i) what is the origin of NI cells in allografts with or without stents? ii) to what (precise) degree do recipient cells contribute to the NI formation? and iii) what is the identity of the NI cells that populate the allograft NI?

## Results

### Model of Allograft Vascular Disease and Stent NI Formation

**Figure 1:**
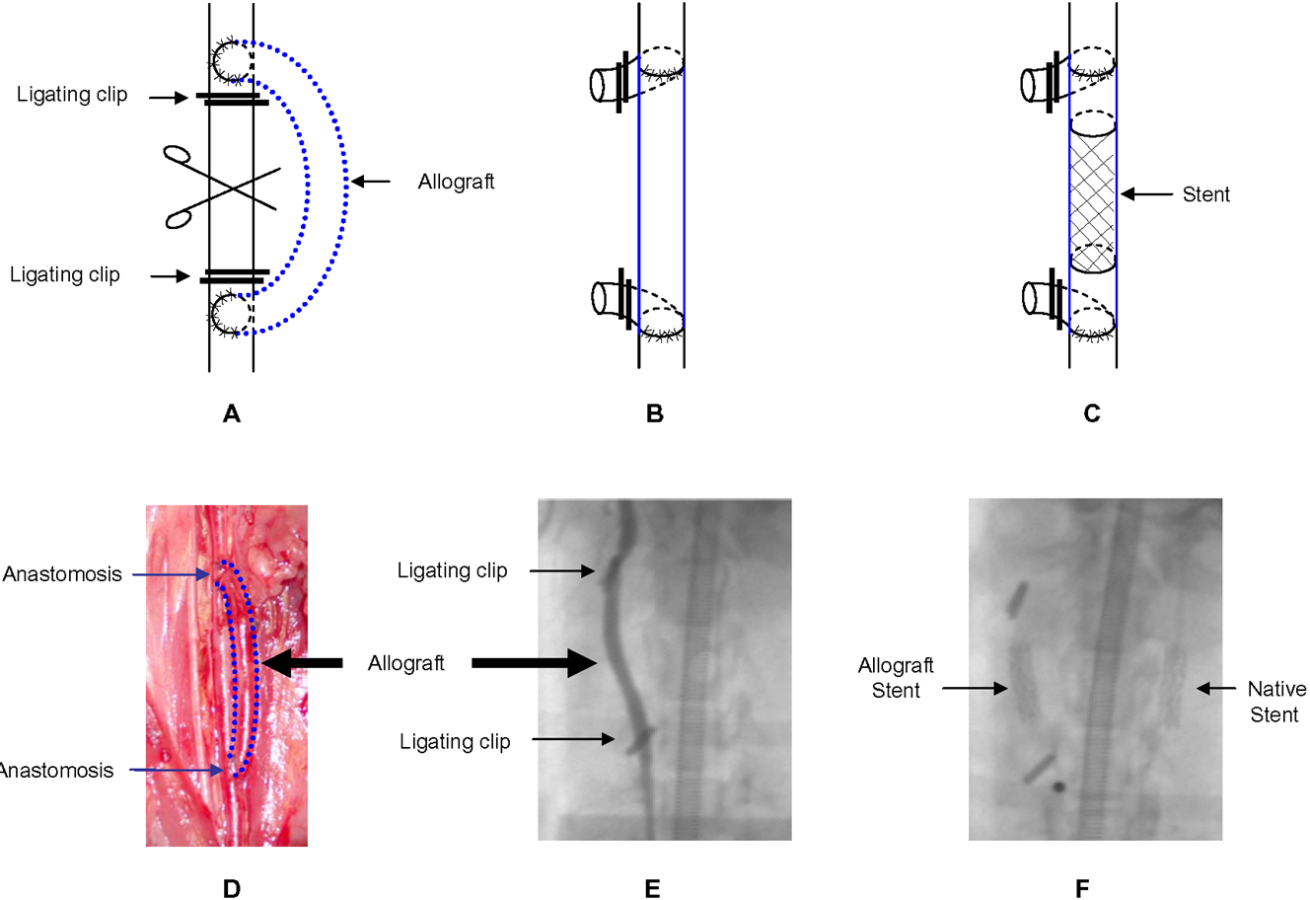
Sex-mismatched rabbit common carotid allograft model. (A–C) Donor carotid artery acting as an allograft to the recipient's common carotid artery. Titanium clips were used to ligate recipient artery. After transplantation the recipient carotid was cut in the middle and the cerebral circulation was restored in the allograft. Three weeks later, a S670 stent (3.0 diameter×12 mm length) was deployed in the middle of the allograft. (D) Photo of microsurgery showing the bypass allograft anastomosed to native common carotid. (E) Angiogram of the allograft after stent implantation. (F) Patent allograft (without contrast dye) showing the radiopaque stent located in the middle of the graft. A stent is also present in the contralateral native carotid artery.

New Zealand White (NZW) rabbits were used for these experiments because their carotid artery is similar in calibre to human coronary arteries and hence suitable for both surgical transplantation and stent deployment. Allografting of a female carotid artery to a male recipient (n = 6), and vice versa (n = 6), was performed (**Figure 1A–F** **, Movie S1 and S2** available online). Two weeks after stent deployment rabbits were euthanized and vascular tissues were harvested for analyses. Total plasma cholesterol levels increased almost fourfold after 5 weeks of ingesting a cholesterol-enriched diet (e.g., baseline: 56.7±2.0 mg/dL and at euthanasia: 200.2±13.2 mg/dL).

### Immunocytochemical and Histomorphological Characterization of NI

**Figure 2:**
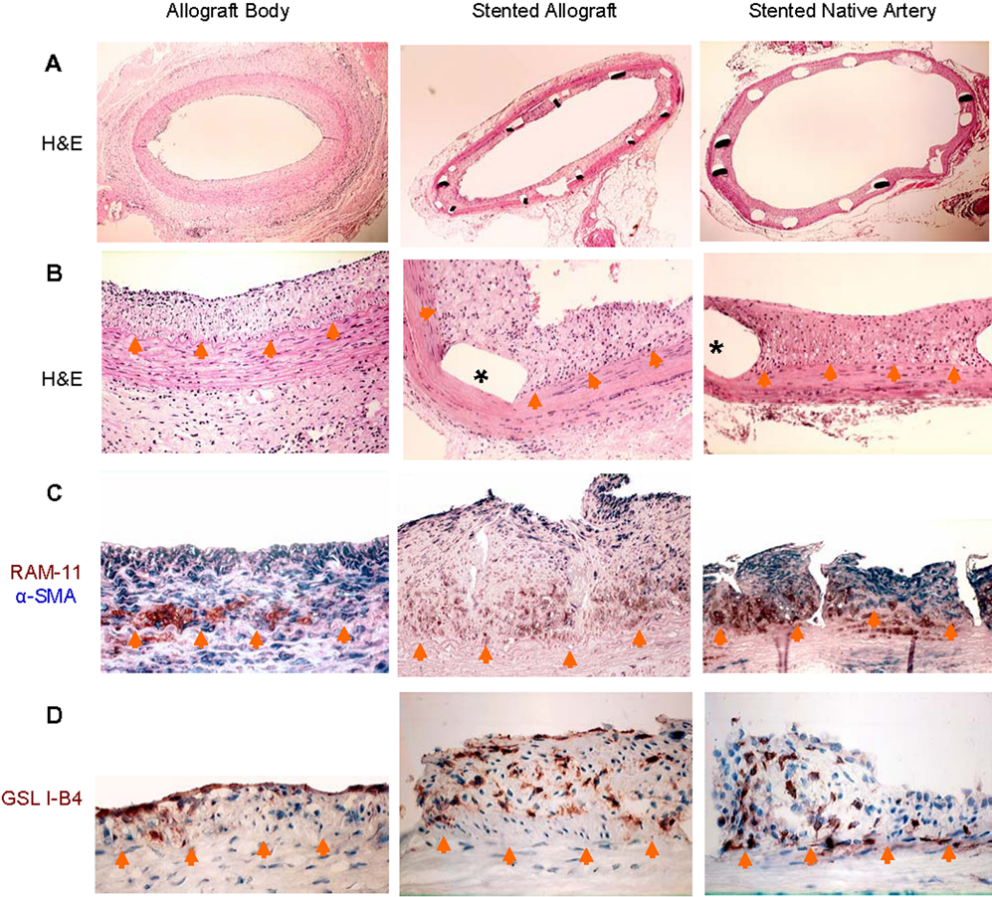
Morphologic and immunohistochemical study of allograft, stented allograft and native artery. A & B: H&E stained tissue section shown at ×20–40 (top row) and ×200 (second row) magnifications with * denoting location of stent struts removed post-mortem. C: Dual labeling for the macrophage marker RAM-11 (brown) and the SMC marker α-SMA (blue) demonstrates similar basal NI location of macrophages with overlying SMCs. Note that larger allograft stent NI has a large number of cells that do not label with either of these cell markers. D: Labeling for the endothelial specific marker GSL I-B4 (brown) shows immunopositive cells within the NI, particularly in the stent NIs of the allograft and native artery. Orange arrow heads delineate the internal elastic lamina.

A modest NI was present in all non-stented and stented allografts segments as well as stented native carotid arteries (**Figure 2A & B**). In non-stented native carotid arteries a NI was absent (data not shown). In non-stented allograft segments the NI largely consisted of an almost equal mix of SMCs and macrophages (**Figure 2C**). As well, a minor infiltration of lymphocytes was present in the allograft NI-consistent with previous descriptions of the early NI phase of ACD[10]. The allograft stent NI was distinct from non-stented allograft segments as well as the stent NI of native carotid arteries. Specifically, in the stent NI of 7/12 allografts more than 50% of the cells failed to label with SMC, macrophage or lymphocyte markers. In the NI of the remaining stented allografts 4 were dominated by macrophages and 1 by SMCs. In contrast, the NI of stented native arteries largely consisted of SMCs with macrophages that were confined to the peri-strut tissue-consistent with our previous description of stent NI formation in native rabbit carotid arteries[18]. Griffonia Simplicifolia Lectin I-isolectin B4 (GSL I-B4) was used to identify cells of endothelial lineage as it is well recognized as a reliable endothelial marker[19], [20]. As expected, the luminal endothelium of non-stented allograft segments was intact and immunolabeled for the endothelial marker GSL I-B4 (**Figure 2D**). However, we were surprised to find scattered within the NI of the non-stented and stented allograft segments, as well as stented native arteries multiple cells that were immunopositive for this endothelial marker–yet did not appear to be part of vasa vasorum (i.e., no organized cellular network encompassing red blood cells).

**Figure 3:**
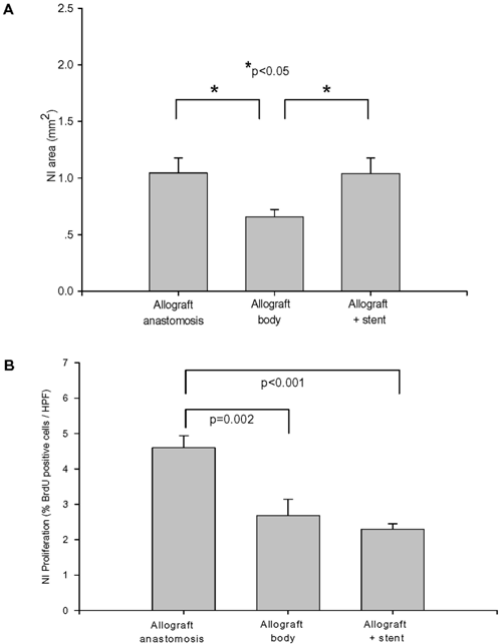
Characterization of NI formation in stented allograft. A: The NI area of the allograft at the site of stent implantation, anastomoses, and the adjacent non-stented allograft body segment, n = 12, *p<0.05). B: Low level of proliferation in the non-stented body of the allograft and stented allograft segments. Proliferation at the anastomoses was higher at the non-stented body of the allograft (n = 12, p = 0.002) or stented allograft (n = 12, p<0.001).

While a modest NI formed in the allografts NI area varied according to the location within the allograft (**Figure 3A**). At the allograft anastomotic sites (the ends of the allograft that were sutured to the carotid artery) the NI area was 59% larger than in the non-stented body of the allograft (1.05±0.13 mm^2^ *vs.* 0.66±0.07 mm^2^, respectively, p<0.05). Interestingly, the area of the NI at the site of stent implantation was 1.04±0.14 mm^2^ and approximately 58% larger than the adjacent non-stented allograft segment (p<0.05). In contrast, the NI generated in response to stenting the contralateral (native) carotid artery was 0.56±0.04 mm^2^ or only 54% of the allograft stent NI area (p<0.01) and similar in magnitude to the non-stented allograft NI found at a distance from anastomotic sites.

As a separate control for this allograft model, we studied a cohort of three rabbits that underwent sex-mismatched carotid artery transplantation but were euthanized 3-weeks post-transplantation (i.e., without stent insertion). A NI was present in all three 3 week old allografts, but was much smaller than that of the 5 week old stented allografts (0.087±0.017 mm^2^ *vs.* 1.04±0.14 mm^2^, p<0.01). The NI cells of the 3 week old allografts consisted mainly of SMCs and macrophages.

To determine the role of proliferation in the NI BrdU immunolabeling was performed (**Figure 3B**). In the non-stented (n = 10) and stented (n = 9) allograft segments the prevalence of proliferating cells was low (2.7±0.5% and 2.3±0.2%; respectively). The proliferation profile at the anastomoses (4.6±0.3%, n = 10) was higher than that of the non-stented and stented allograft sites (p = 0.002 and p<0.001; respectively). In the native carotid artery (no stent) proliferation was virtually absent, but at the site of stent insertion 1.6±0.3% of cells were proliferating–a frequency similar to that found in non-stented or stented allografts and certainly lower than that detected at the allograft anastomoses (p<0.001).

### Origin of Stent NI Cells

**Figure 4:**
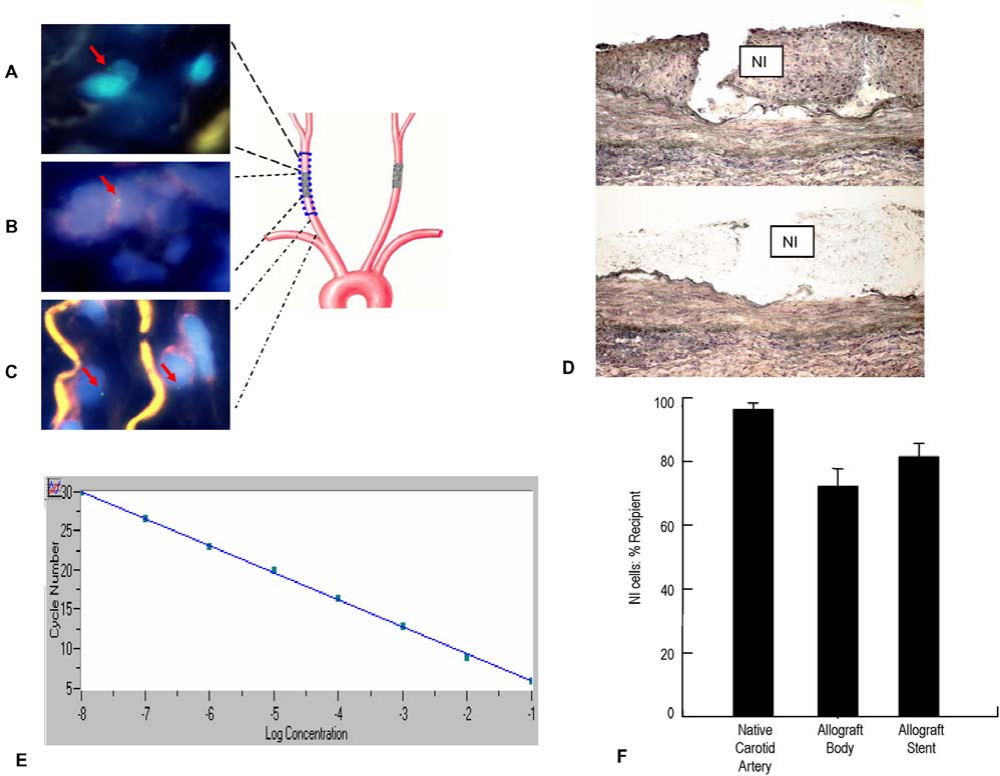
A–C: FISH hybridization with an SRY specific probe in the NI of a female-to-male allograft with or without stent. Blue areas show DAPI staining of nuclei with green labeling of Y chromosomes (highlighted with red arrows). The yellow color is due to auto fluorescence of elastic fibers. Male cells (recipient origin) were detected in NI of female donor allograft without (A) or with stent (B). Male native carotid artery medial cells are SRY positive (C) and were used as positive controls. D: Arcturus HistoGene stained allograft arterial wall before and after NI microdissected by LCM. NI = neointima. E: Sample Q-PCR standard curve for GAPDH. F: Percent contribution of recipient cells to the NI in allograft body, allograft stent, and native vessels.Bars represent means±SEM.

To assess the contribution of the recipient's cells to stent NI formation we employed two techniques. First, by using Fluorescent *in situ* hybridization (FISH) for the Y chromosome male recipient cells were detected in the NI of the non-stented segment of a female-to-male allograft (**Figure 4A**), the NI in the allograft stent (**Figure 4B**), and in the media of the proximal native artery (**Figure 4C**). Similar to other reports[17], [21] the sensitivity of FISH to detect male cells was limited (e.g., we detected only 20.0±2.6% of positive cells in pure male tissue)–despite adequate hybridization as reflected by parallel use of a control probe specific for histone-H3 (data not shown).

Second, Laser capture microdissected (LCM) was used to precisely isolate stent NI tissue (**Figure 4D**) and quantitative polymerase chain reaction (Q-PCR) assessment of Y chromosome copy number relative to that of an autosomal gene was employed to determine the percentage of host or donor cells involved in NI formation. The NIs ranged in area from approximately 0.48–1.48 mm^2^, and had an average cell density of 6,085 cells/mm^2^; hence between 2,434 and 9,005 cells were sampled on each tissue slide using LCM. **Figure 4E** shows the linearity of a sample standard curve for GAPDH. Both the sex-determining region Y (SRY) and GAPDH reactions were linear over a one million fold range for the concentration of the standard plasmid. Furthermore, the correlation coefficient for SRY and GAPDH were excellent (r^2^ value range: 0.99–1.00 and 0.98–1.00; respectively). Hence, using this technique and the appropriate calculations we were able to determine the origin of cells in the NI of both male and female arteries. Examination of nine native carotid artery samples (5 female and 4 male) revealed that 96.5±6.6% of the cells were of recipient origin (**Figure 4F**). The contribution of recipient cells to the allograft NI in non-stented segments (n = 9) was 72.1±5.7% of cells. In the larger NI of the allograft stents (n = 10) 81.5±4.2% of the cells were of recipient origin. Therefore, by inference approximately 27.9% of the non-stented allograft NI cells originated from the allograft, while only 18.5% of the stent NI cells in the allografts were derived from the vessel wall. Given the relatively low proliferation profile in the allograft NI, these data suggest that host cell engraftment plays a major role in allograft NI formation.

### Differentiation of Circulatory Cells: In Vitro and In Vivo

**Figure 5:**
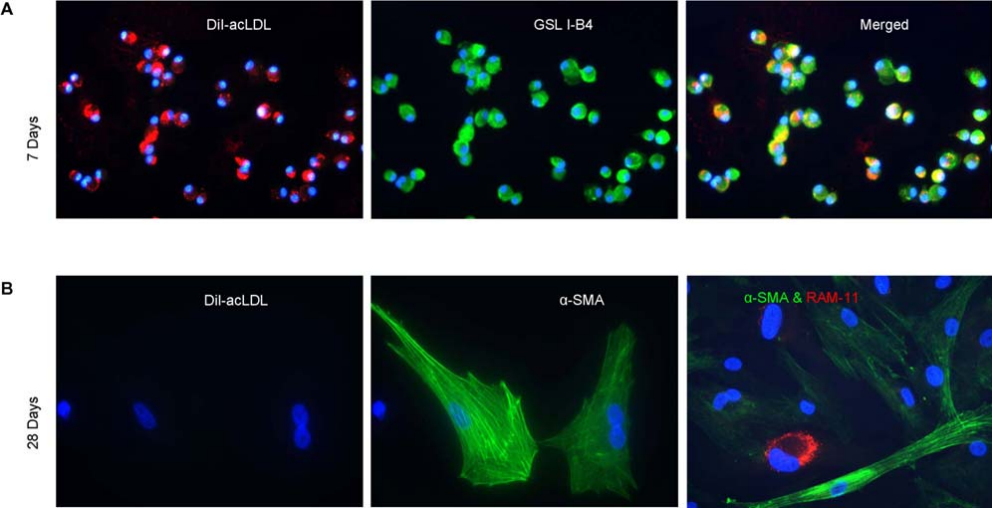
Differentiation of circulatory cells *in vitro.* A: Rabbit PBMCs maintained in culture for 7 days: DiI-acLDL labeling (red, left panel); GSL I-B4 labeling (green, middle panel); Merged image showing both DiI-acLDL uptake and GSL I-B4 labeling in individual cells (yellow, right panel). B: Rabbit PBMCs after 28 days in culture: DiI-acLDL labeling is absent (red, left panel), α-SMA expression is evident (green, middle panel), and some cells that do not express α-SMA express the macrophage marker, RAM-11 (red, right panel). For both A and B: blue DAPI nuclear counterstain and ×400 magnification.

To test whether the GSL I-B4 immunopositive NI cells (described above) may be host-derived cells that differentiate from an endothelial to a SMC or monocyte/macrophage lineage we cultured rabbit peripheral blood mononuclear cells (PBMCs) on fibronectin-coated plates in EGM-2 media. Similar to human PBMCs maintained in culture for seven days[22] the adherent rabbit PBMCs on day 7 in culture were positive for both DiI-acLDL uptake and GSL I-B4 labeling but negative for both α-smooth muscle actin (SMA) and RAM-11 immunolabeling (**Figure 5A**). However after 28 days the majority of cells expressed α-SMA or RAM-11 but did not show DiI-acLDL uptake or GSL I-B4 immunolabeling (**Figure 5B**). Hence, these *in vitro* data suggest that PBMCs are capable of transdifferentiating from an EC lineage to either SMCs or monocyte/macrophages.

**Figure 6:**
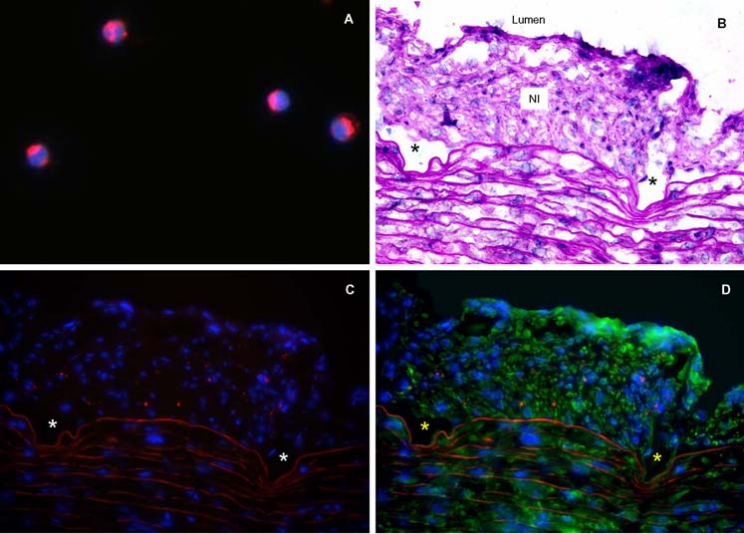
Differentiation of circulatory cells *in vivo.* A: PBMCs labeled *in vitro* with PKH26 dye (red) and DAPI nuclear counterstain (blue); magnification: ×1000. These cells were injected into the carotid artery immediately after stent insertion. B: Cross section of native rabbit carotid artery 14 days post stent implantation (H&E stain,). C: PKH26-labelled PBMCs (red) were detected in the NI of stented artery. D: Some PKH26-labelled PBMCs (red) were also immunopositive for α-SMA (green). For panels B–D: arrows or autofluorescence delineate the internal elastic lamina; *denote sites of manually removed stent struts; magnification: ×400.

Similarly, to directly determine if circulatory cells engraft stent NI, rabbit PBMCs tagged *ex-vivo* with the fluorescent dye PKH26 (**Figure 6A**) were selectively injected into native carotid arteries immediately after stent implantation. Fourteen days after stent implantation PKH26-tagged cells constituted 9.0±0.4% of stent NI cells per high power field (HPF) (**Figure 6B–D**).

## Discussion

ACD ultimately leads to failure of cardiac transplants and is difficult to manage due to its relative refractoriness to immune modulation and/or a variety of revascularization procedures, including percutaneous coronary stent insertion. Distinct from plaque rupture in atherosclerotic native coronaries, progressive NI accumulation is the ultimate cause of ACD as well as allograft ISR[1]. In order to improve our understanding and treatment of ACD and allograft ISR, we developed a novel sex-mismatched rabbit model of carotid artery grafting in order to quantitatively study the contribution of host *vs.* donor cells during NI formation. Using both *in situ* hybridization for the Y chromosome and Q-PCR for the Y chromosome on NI tissue isolated with the aid of LCM we show that host derived cells play a dominant role in the genesis of allograft NI formation. Specifically, 5 weeks after allografting 72.1% of allograft NI cells are of host origin. Moreover, when a stent is implanted in a 3 week old allograft and the resulting NI is examined 2 weeks later we note that 81.5% of the allograft stent NI cells are host derived.

While a number of human studies unquestionably demonstrate the presence of host derived cells in allograft NI formation[12]–[17], considerable debate lingers in the experimental literature regarding this phenomenon. In part, it can be argued that the experimental models are poor mimics of the human condition, and while undoubtedly true, this sweeping statement does not do justice to the plethora of excellent experimental studies in this field. Space does not permit a balanced discussion of all of these studies, hence interested readers are referred to two excellent reviews by Hillebrands *et al*.[10], [23].

In the current study we used two techniques to detect the Y chromosome: FISH on arterial cross sections and Q-PCR on sections of NI carefully isolated with LCM. While the sensitivity of FISH for the Y chromosome is widely recognized to have limited sensitivity (e.g., varying between approximately 20–50%) [17], [21] this technique is useful for localization reasons. Quite the opposite, Q-PCR is exquisitely sensitive for quantifying the percentage of male tissue in chimeric systems using Y chromosome specific amplicons (e.g., Y chromosome detection thresholds as low as 0.125%–equivalent to 6 copies of the Y chromosome) [24]–[27]. We examined a far larger number of allografts than the most relevant previous study whereby the investigators limited their cell sampling protocol to cells that were α-SMA immunopositive and derived their conclusions from the analyses of only 21 nuclei harvested from 3 allografts[28]. In contrast, we performed LCM on a total of 12 allografts (6 male to female, and 6 female to male), with analyses performed at 2 specific subsegments within each graft (i.e., unstented body of graft, and stented mid-segment). As well, using Q-PCR protocol with remarkable linearity for the amplification reaction, we do not rely simply on the presence or absence of an amplicon. Thus, while we acknowledge the pioneering work by other investigators in this field, particularly Hillebrands and colleagues, our work expands upon the initial studies and provides a large data set to draw quantitative conclusions from.

Undoubtedly, the NI of the allografts in our study is the result of both the response to stent implantation and the alloimmune features of vasculopathy; however, this is no different from the cardiac transplant recipient with ACD who undergoes coronary stenting. Stenting ultimately results in increased NI formation that cannot be explained by excessive proliferation. Actually, the proliferation rates in stented or non-stented allografts were similarly low and comparable to that of the stented native arteries–observations that are consistent with our previous study of the response to stent implantation in rabbits[18].

To further demonstrate that rabbit circulatory cells can populate and differentiate into vascular cells within the NI of stents we performed two additional experiments. First, we demonstrated that PBMCs from rabbits can differentiate *in vitro* and at day 7 express markers consistent with an endothelial lineage (DiI-acLDL uptake, GSL I-B4 labeling). By 4 weeks *in vitro* the same cells no longer express these endothelial cell markers, but instead showed features consistent with either SMCs or macrophages (e.g., immunolabeled for α-SMA or RAM-11; respectively). Second, when PBMCs are tagged with the fluorescent PKH26 dye and injected at time of native carotid artery stent insertion, they constitute a surprisingly high percentage (9%) of the stent NI cell population 14 days later.

Perhaps the most intriguing observation from the current study is the detection of cells that express the endothelial lineage marker GSL I-B4[19], [20] within the NI of both non-stented and stented allograft segments, as well as stented native arteries. *In situ*, these cells are morphologically similar to NI SMCs, as they are surrounded by an extracellular matrix and do not appear to be part of an organized vasa vasorum. Certainly, in the porcine model of stent neointimal formation, SMCs almost exclusively populate the neointima, thereby raising the possibility that these GSL I-B4 cells may be unique to the rabbit model[29]. While the involvement of cells expressing other endothelial markers in experimental lesions formation is not novel [30], [31] to our knowledge, this is the first report of GSL I-B4 positive cells within the NI of stented arteries. Given our current data as well as that from other laboratories[32]–[34] showing the differentiation of PBMCs to either endothelial cells, SMCs or macrophage phenotypes, it is tempting to speculate that GSL I-B4 positive NI cells are a form of circulatory progenitor cells that later differentiates *in vivo* into a SMC or macrophage phenotype. It is less likely that these cells eventually constitute vasa vasorum within the stent NI because although neovascularization of atherosclerotic lesions is common, vasa vasorum are exceedingly rare in ISR lesions[35].

Finally, there are limitations to our studies. First, it must be remembered that we detected host-derived NI cells at a relatively early interval in lesion development and it is unclear if these host-derived cells survive and participate in mature lesion formation. While Religa and colleagues [36] noted a similar frequency of host-derived SMCs in 8 week old allografts, Bentzon and colleagues [37] found only locally derived cells at 20 and 32 week old after allografting. Hence, it is possible that the early engraftment of host-derived cells (perhaps facilitated by apoptosis of local vessel wall cells) is later replaced by a regrowth of local cells that are stimulated by immune-mediated inflammation. A second question relates to the prospect that host cells from the native carotid artery anastomoses migrated inward to colonize the allografts. Our rabbit model used isotransplanted carotid segments that are quite long (30 mm). Given that each stent was 12 mm long and placed in the middle of the allograft, vascular cells from the adjacent native carotid artery would have to migrate at least 9 mm in a period of two weeks to reach the margins of the stent. It is unlikely that two weeks is sufficient time for inward migration of cells from the anastomoses to populate the stent NI. Moreover, in our study the NI of the allograft body between either anastomosis and the stent is approximately 50% smaller than that of the stent itself. Therefore, if inward migration of mature carotid artery cells contributed to stent NI formation, it is surprising to note that the intervening allograft body shows a “gap” in the NI area relative to that of the more centrally located stented segment. Third, the engraftment of *ex vivo* PKH26-labeled cells into the stent NI was remarkably high, and perhaps reflects modifications of these cells that occurred while in culture (e.g., upregulated expression of integrins). Hence, we must cautiously interpret the magnitude of this observation, yet can acknowledge that this information is supportive of the concept that blood derived cells can directly participate in stent NI formation. Finally, one must ask why were all of the NI cells not blood borne? Perhaps one clue might come from elegant studies performed decades ago that clearly demonstrated the role of tissue wall hypoxia in the genesis of the NI. Essentially, the vasa vasorum of carotid arteries arise from terminal branches of adjacent arteries (e.g., ophthalmic)[38]. Hence, the harvesting of these arterial allografts ultimately results in disruption of the vasa vasorum and leads to vessel wall hypoxia–a phenomenon previously shown to induce atherosclerotic lesions[39]. Therefore, it begs the question could this hypoxic stimulus be a confounding factor that prompts NI expansion by endogenous SMCs that, in effective, partially minimize the involvement of blood borne (circulatory) cells?

In summary, our sex-mismatched carotid allograft rabbit model provides a novel method to study NI formation in ACD and the response stent implantation in allografts. Our findings suggest that blood-borne, host-derived progenitor cells–including cells of endothelial lineage–are important contributors to NI formation in both these lesions. Hence, modulating the mobilization, homing, and differentiation of host derived circulating cells may provide a new therapeutic target in prevention of ACD and stent NI formation in allografts.

## Materials and Methods

A detailed description of the methodology (Materials and Methods S1) is provided as supporting information.

### Sex-Mismatched Carotid Allograft Model

Animal procedures were carried out with the approval of the University of Ottawa Animal Care Committee and followed the guidelines of the Canadian Council on Animal Care. Carotid artery transplantations were performed between gender-mismatched NZW rabbit siblings (2.5–3.0 kg, Charles River Laboratories, Quebec). Six male and six female rabbits were recipients of sex-mismatched carotid allografts. Beginning one week prior to transplantation the rabbits were fed a 0.3% cholesterol diet (Harlan Teklad, Madison, WI) that was continued till euthanasia. Under general anesthesia, the donor carotid arterial segment (2.5–3.0 cm long) was harvested and the donor allowed to recover for subsequent transplantation or euthanized if donating a second carotid artery. The recipient rabbit was heparinized (175 IU/kg), the right carotid artery exposed, cross-clamped over a 3–3.5 cm segment and incised longitudinally. The harvested donor carotid arterial allograft was anastomosed to the segment end-to-side with interrupted 8-0 prolene sutures. Circulation was restored and vigorous pulsations in the allografted carotid artery without leaking confirmed successful transplantation. Recipients were then allowed to recover for three weeks before a cobalt chromium stent (S670, 3.0×12 mm, Medtronic AVE, Inc., Santa Rosa, CA) was deployed at 6 atmospheres in the middle of the allograft (experimental). As well, a second (control) stent was deployed in the contra-lateral native carotid artery. Immunohistochemistry was performed on tissue sections in order to detect the presence of macrophages, SMCs, T cells, cells of endothelial lineage (GSL I-B4 immunopositive), as well as proliferating cells. FISH was performed with a specific probe for the Y chromosome (SRY). LCM intimal tissue was collected, genomic DNA was isolated and subjected to Q-PCR for SRY and GAPDH genes in order to asses Y chromosome copy number relative to that of an autosomal gene, and therefore, determine the percentage of host or donor cells involved in NI formation.

### Differentiation of PBMCs *in vitro* and *in vivo*

Differentiation of rabbit PBMCs was examined *in vitro* using previously described methodologies[40]. To track the *in vivo* fate of circulatory cells, 1.75×10^7^ of the rabbit's own PKH26 labeled PBMCs (Sigma)[41]–[43] were selectively re-infused into each carotid artery immediately post-stent insertion. One S670 stent was deployed per native carotid artery and 14 days later the rabbits were euthanized and the stented arteries (n = 8) were harvested. The maximum percentage of PKH26 labeled cells per HPF was determined.

## Supporting Information

Movie S1 Angiogram of stented carotid allograft
(2.42 MB MPG)

Movie S2 Angiogram of stented native carotid artery
(2.00 MB MPG)

Materials and Methods S1 (0.07 MB DOC)
