# Supplementary material for: Contribution of Recipient-Derived Cells in Allograft Neointima Formation and the Response to Stent Implantation
Source: PLoS One. 2008 Mar 26;3(3):e1894. doi: 10.1371/journal.pone.0001894 (PMC2267220; doi:10.1371/journal.pone.0001894)
Supplement: Materials and Methods S1 — (0.07 MB DOC) [file pone.0001894.s003.doc]

**Supplemental Materials and Methods**

**Medication**

Surgical procedures were performed under general anesthesia with ketamine [25 mg/kg, intramuscularly (i.m.)], medazolam (2–4 mg/kg, i.m.), and isoflurane (via an endotracheal tube). To mimic clinical practice, rabbits were given a 4 mg/kg loading dose of clopidogrel bisulphate (Sanofi-Synthelabo, Montreal) one day prior to the stenting procedure and 1 mg/kg/day until euthanasia. At the outset of the stenting procedure, an intravenous heparin bolus (125 U/kg) was administered. Furthermore, to limit stent thrombosis, acetylsalicylic acid (10 mg/kg,) was given 3 days a week beginning 3 days after transplantation until euthanization. The stented allografts and native carotids were harvested 2 weeks post stenting. Allograft rabbits were fed a 0.3% cholesterol diet (Harlan Teklad, Madison, WI), that began 1 week before the allograft surgery.

**Tissue Harvest and Quantitative Histomorphologic Analyses**

Fourteen days after stent implantation, animals were euthanized using intravenous Euthanyl (Schering-Plough, Quebec), and the stented allografts and contralateral stented native carotid arteries were harvested. All animals received BrdU (50 mg/kg, *i.v.* Sigma, St. Louis, MO) one hour before euthanasia to allow immunolabeling of proliferating cells. The allografts were divided into 3 anatomic segments: the anastomoses, the allograft body that contains the stent and the intervening segment of allograft body that was not stented. After overnight fixation in 10% neutral buffered formalin (NBF), a sub-segment of tissue was opened longitudinally and used for immunohistochemistry. As well, hematoxylin/eosin (H&E)-stained slides were generated for morphometric analyses using computer-assisted digital imaging (Image-Pro Plus, Media Cybernetics, Silver Spring, MD) to quantify the NI area. For each segment, 6 subsegments of tissue (each separated by 30 µm) were studied using 5 µm thick tissue sections. For stented tissue segments, one sub-segment of tissue was embedded in methylmethacrylate after overnight fixation with 10% NBF, before 5 µm thick cross sections were cut with a D-Profile tungsten carbide knife (Delaware Diamond Knives Inc., Wilmington, DE). Otherwise, stented segments were opened longitudinally and metal stent struts were manually removed prior to either fixation in 10% NBF or freezing in OCT (Miles Inc., Elkhart, IN).

**Immunohistochemistry**

Immunohistochemistry was performed on paraffin-embedded and fresh-frozen specimens. The following primary antibodies were used: RAM-11 (titre 1:50; Dako, Mississauga, Ontario) for macrophages; an antibody directed against α-smooth muscle actin (α-SMA, Sigma) to identify smooth muscle cells; an anti-CD43 antibody (titre 1:50; Serotec) for T cells; and an anti-BrdU antibody to immunolabeling actively proliferating cells (titre 1:50; Dako). Biotinylated Griffonia Simplicifolia Lectin I - isolectin B4 (titre 1:600; GSL I-B4, Vector) was used for labeling endothelial cells. After incubating with the unconjugated primary antibody, a species-specific biotinylated or fluorescent-conjugated secondary antibody was applied. Subsequently, samples were incubated with an avidin-biotin peroxidase or alkaline phosphatase complex (VECTOR) and visualization with either 3,3-diaminobenzidine (Sigma) or 5-bromo,4-chloro,3-indolylphosphate / nitrobluetetrazolium (BCIP/NBT) substrate (Sigma). Slides were counterstained with either hematoxylin or orcein. Fluorescent immunolabeled specimens underwent nuclear counterstaining with 4'-6-Diamidino-2-phenylindole (DAPI, Vector). Anti-BrdU immunolabeled proliferating cells in the NI were counted and expressed as a percentage of the total number of NI cells per high power field (HPF, magnification: ×400). For these proliferation studies 3 HPFs were counted per arterial cross-section and 3 cross-sections were examined per arterial subsegment (i.e., body, anastomosis, allograft stent, native stent) – with at least 9 arteries examined per experimental group (i.e., male to female and female to male allografts). To determine the predominant NI cell type a low power (×40) magnification that captured the entire arterial cross section was used to estimate if one cell type occupied more than 50% of the NI area. In some arterial sections a mixture of cell types or no cell type dominated the NI cell population. For the histological studies 12 arteries were analyzed in each study group, using 6 arterial cross sections (25 µm apart) for each location within the allograft or stented native artery.

**Fluorescent In Situ Hybridization (FISH)**

A 200 bp SRY probe was generated for FISH on paraffin embedded tissue sections. Briefly, a fragment of the SRY gene was amplified from rabbit genomic DNA using the primers: SRYfishfwd AACGCGTTCATGGTGTGGTC and SRYfishrev GAGGTCTGTACTTGTAGTCC. The amplicon was cloned, sequenced and isolated using standard protocols. The SRY probe was then generated by random priming with digoxigenin-dUTP (Roche, Germany). Tissue sections were deparaffinized, dehydrated, and dried. Samples were then denatured by incubation 72°C for 10 minutes and the labeled SRY DNA probe added. Hybridization was allowed to proceed at 42°C overnight and the samples were washed the next morning. Tissue sections were then incubated with anti-DIG-fluorescein at 37 °C for 1hr. Samples were mounted in Vectashield Mounting Medium with DAPI and examined by fluorescent microscopy.

**Laser Capture Microdissection (LCM)**

LCM was performed with the Arcturus PixCell II system (Arcturus Engineering, Mountain View, CA). Briefly, frozen tissue sections were stained with HistoGene Staining Solution and the NI were dissected onto microdissection caps using a low-energy, infrared laser (pulse power: 50 mW, pulse width: 10 ms, laser spot size: 30 μm) [1,2].

**Molecular Cloning and Plasmid Preparation for Q-PCR**

Briefly, plasmids containing SRY and GAPDH fragments were generated for Q-PCR analysis. Primer/probe combinations and target sequences were selected using PrimerQuest software (<http://scitools.idtdna.com/Primerquest/>). A 186bp fragment of GAPDH was generated using the following primers: GAPDHfwd CGCCTGGAGAAAGCTGCTAAGTAT and GAPDHrev GCTTCACAAAGTGGTCATTGAGGG. Similarly, a 201 bp fragment of SRY was generated using the following primers: SRYfwd GAAACTCAGACATCAGCAAGCAGC and SRYrev AGGTCTGTACTTGTAGTCCGGGTA. Fragments were cloned into the pGEM-T vector, sequenced, and isolated using the PhasePrep BAC DNA kit (Sigma). The plasmids were then linearized, purified, and diluted over a 10^8^ fold range to generate standard curves for quantitative PCR analysis. Target plasmid copy number was calculated using the molar concentration, as determined by UV spectroscopy on a GeneQuant Pro (Biochrom, England), and the molecular mass of the plasmid and the insert. Molecular mass was ascertained using sequence data and Genamics Expression (ver. 1.1) genomics software package.

**Quantitative PCR Protocol**

Genomic DNA was isolated from the caps (up to 5.4 × 10^4^ cells per sample) with the QIAamp DNA Micro Kit (QIAGEN). Q-PCR reactions were performed using the Roche LightCycler Q-PCR system and analyzed using the Roche Molecular Biochemicals LightCycler software package (ver. 3.5). To minimize variability and ensure reproducibility, standard curves were obtained with each set of reactions and standard or experimental samples were performed in triplicate. Both LCM tissue and SRY/GAPDH standard plasmid DNA were isolated as previously described. Primers used were GAPDHfwd/GAPDHrev and SRYfwd/SRYrev for GAPDH and SRY respectively. Probes were designed using the PrimerQuest software and were as follows: GAPDHprobe AAGAAGGTGGTGAAGCAGGCATCCGAGGGC; SRYprobe ATGGCCATTCTTCCAGGAGGCGCAAAGACT. Probes were 5’ 6-FAM labeled and 3’ TAMRA modified. All primers and probes were purchased from Alpha DNA (Montreal, Quebec). The Q-PCR reaction mixture contained template DNA, 1× QuantiTect Probe PCR (Qiagen), 0.5 µmol/L fwd primer, 0.5 µmol/L rev primer, and 0.2 µmol/L of gene specific probe. Cycling conditions consisted of 95^o^C for 15 minutes, 95^o^C for 15 seconds then 60^o^C for one minute (50 cycles) and 40^o^C for 30 seconds. LCM sample target copy number was determined by comparison to a known standard curve and then expressed as the mean of three replicates. As accuracy was paramount, standard curves were run with each reaction and samples were repeated in triplicate with the mean copy number for SRY and GAPDH sequences used to calculate the final ratio. All samples were assessed in a randomized and blinded fashion.

**Calculation of Donor vs. Recipient Cellular Contribution to NI Formation**

Amplicons of SRY or GAPDH were cloned into pGEM-T vector using standard cloning techniques. Using a Genequant DNA/RNA calculator (BioRad), the concentration of DNA was determined (n=6) and the mean value obtained used in subsequent calculations. Stock solutions were then diluted over a 10^8^ fold range. Four μl of stock solution was utilized in each reaction used to generate the standard curve. Molar mass of plasmid and amplicon fragments was determined using Genamics Expression (v1.1). Copy number was calculated using the formula below:

Copy # = (DNA concentration) × (volume in reaction) × (Avogadro’s number) / _molar mass of plasmid+amplicon_

Sample Calculation: Copy # = (695.5 × 10^-9^ g/μL) × (4 μL) × (6.022 × 10^23^ molecules/mole) / 1.955 g/mole = 8.6 × 10^17^ molecules

Standard curves were expressed as copy number and outputs from the Roche Molecular Biochemicals LightCycler software package were expressed in copy number. Percent male tissue was calculated using the formula below:

% male tissue = ^200^/_(GAPDH copies/SRY copies)_

Sample Calculation: % male tissue = ^200^/_(40,000/10,000)_  = 50% male tissue

**Differentiation of PBMCs *in vitro* and *in vivo***

Differentiation of rabbit PBMCs was examined *in vitro* using previously described methodologies[3]. Briefly, PBMCs were isolated by density gradient centrifugation with Histopaque-1077 (Sigma). Cells were washed twice with Hank’s balanced salt solution (HBSS). Fibronectin (Sigma) coated six-well plates were then seeded with 5 × 10^6^ PBMCs/well. Culture media consisted of endothelial cell medium (EGM-2, Cambrex, East Rutherford, NJ) supplemented with SingleQuots (Cambrex). Seven and 28 days after plating, non-adherent cells were removed and adherent cells were washed three times with HBSS before being incubated with DiI-acLDL (10 μg/ml, Molecular Probes, Eugene, OR) for 1 hour at 37^o^C. Subsequently, cells were fixed with Cytofix Buffer (Becton-Dickinson, Franklin Lakes, NJ) before incubation with a FITC conjugated GSL I-B4. Following incubation, plates were washed three times with HBSS. EPCs were defined by the uptake of acLDL (acLDL^+^) and GSL I-B4 binding. For double immunolabeling the first primary antibody directed against α-SMA was detected with a FITC-conjugated anti-mouse IgG (Vector Laboratories), and the second primary antibody directed against RAM-11 was detected with a Texas-Red conjugated anti-mouse IgG (Vector Laboratories). Cell nuclei were counterstained with DAPI in the Vectashield Mounting Media (Vector Laboratories).

To track the *in vivo* fate of circulatory cells, PBMCs were isolated by density gradient centrifugation of rabbit blood and labeled with the PKH26-GL red fluorescent cell linker kit (Sigma)[4-6]. After deploying one S670 stent in each carotid artery of four rabbits (using the techniques described above), 1.75×10^7^ of the rabbit’s own PKH26 labeled PBMCs were selectively re-infused into each carotid artery immediately post-stent insertion. After 14 days the rabbits were euthanized and the stented arteries (n=8) were harvested and processed, as described above. For each carotid artery six serial cryosections of 5 μm thickness, obtained at 50 μm intervals, were counterstained with DAPI in Vectashield Mounting Medium (Vector) and analyzed using fluorescent microscopy (Olympus BX60 microscope, Olympus America Inc, Center Valley, PA). The maximum number of PKH26 staining cells in one HPF was determined, and this result was divided by the total cell number in order to yield the (maximum) percentage of PHH26 cells per HPF.

**Statistics**

All data are presented as mean±SEM. A one-way ANOVA was used for multiple comparisons between groups. Unpaired *t* test was used for comparisons between two groups. Significance was defined by a *p* value<0.05.

Reference List

1. Emmert-Buck MR, Bonner RF, Smith PD, Chuaqui RF, Zhuang Z, et al. (1996) Laser capture microdissection. Science 274: 998-1001.

2. Bonner RF, Emmert-Buck M, Cole K, Pohida T, Chuaqui R, et al. (1997) Laser capture microdissection: molecular analysis of tissue. Science 278: 1481,1483.

3. Hristov M, Erl W, Weber PC (2003) Endothelial Progenitor Cells: Isolation and Characterization. Trends in Cardiovascular Medicine 13: 201-206.

4. Werner N, Junk S, Laufs U, Link A, Walenta K, et al. (2003) Intravenous transfusion of endothelial progenitor cells reduces neointima formation after vascular injury. Circ Res 93: e17-e24.

5. De Leon H, Ollerenshaw JD, Griendling KK, Wilcox JN (2001) Adventitial Cells Do Not Contribute to Neointimal Mass After Balloon Angioplasty of the Rat Common Carotid Artery. Circulation 104: 1591-1593.

6. Jabs A, Moncada GA, Nichols CE, Waller EK, Wilcox JN (2005) Peripheral Blood Mononuclear Cells Acquire Myofibroblast Characteristics in Granulation Tissue. J Vasc Res 42: 174-180.
